# Associations between 100% Orange Juice Consumption and Dietary, Lifestyle and Anthropometric Characteristics in a Cross-Sectional Study of U.S. Children and Adolescents

**DOI:** 10.3390/nu11112687

**Published:** 2019-11-06

**Authors:** Junichi R. Sakaki, Melissa M. Melough, Jing Li, Rulla M. Tamimi, Jorge E. Chavarro, Ming-Hui Chen, Ock K. Chun

**Affiliations:** 1 Department of Nutritional Sciences, University of Connecticut, Storrs, CT 06269, USA; 2 Department of Statistics, University of Connecticut, Storrs, CT 06269, USA; 3 Harvard Medical School, Department of Epidemiology, Harvard T.H. Chan School of Public Health, Channing Division of Network Medicine, Brigham and Women’s Hospital, Boston, MA 02115, USA; 4 Department of Nutrition and Epidemiology, Harvard T.H. Chan School of Public Health, Boston, MA 02115, USA

**Keywords:** 100% orange juice, fruit juice, children, adolescents, obesity, anthropometry, weight, height

## Abstract

Concerns about orange juice’s sugar content have spurred discussions regarding its potential contributions to childhood obesity. The objective of this study was to evaluate the association between 100% orange juice (OJ) consumption and dietary, lifestyle and anthropometric characteristics in children and adolescents. Baseline anthropometric, dietary and lifestyle data from the Growing Up Today Study I (GUTS I) and GUTS II were collected via self-reported questionnaires from 26,554 participants. The mean values of these variables were then computed for children grouped by categories of OJ consumption, and linear trend testing was used to determine whether OJ consumption was linearly related to these variables. Multivariate adjustment modeling was used to calculate odds ratios of being obese or overweight/obese by OJ consumption. Among both boys and girls, OJ intake was positively associated with height, height-for-age z-score (HAZ), intakes of total energy, total energy excluding OJ, fruits and non-starchy vegetables and physical activity. BMI, BMI z-score and BMI-for-age percentile did not differ by OJ consumption. After adjustment for cohort, age, race, total energy intake without OJ, physical activity and screen time, prevalence of overweight/obesity significantly decreased by OJ intake in boys compared to non-consumers (odds ratio (OR) and 95% confidence interval (CI)): 1.17 (1.02, 1.33) for 1–3 glasses per month, 1.11 (0.98, 1.26) for 1–6 glasses per week, 1.00 (0.85, 1.18) for 1 glass per day, 0.91 (0.73, 1.13) for ≥1 glass per day, *p*-trend = 0.0403). Prevalence of obesity similarly decreased by OJ intake in boys. Prevalence of overweight/obesity and obesity did not significantly differ by OJ intake in girls. The results indicate that children consuming more OJ tended to practice healthier dietary and lifestyle habits without increased prevalence or odds of obesity or overweight.

## 1. Introduction

One-hundred percent orange juice (OJ) is one of the most popular types of juice [1], and it is widely available and nutrient-dense. A typical 6 fl oz serving of OJ provides over 100% of the recommended dietary allowance for vitamin C and 85 kcal [2]. OJ is also a good source of other micronutrients such as potassium, folate and thiamin, and is commonly fortified with calcium and vitamin D [3]. Data from the National Health and Nutrition Examination Survey (NHANES) 2003–2006 indicated that children and adolescents consuming greater amounts of OJ were more likely to meet the estimated average requirements for vitamins A, C and D as well as folate, magnesium and calcium [4,5]. In addition, OJ is the second largest contributor to flavonoid intake in the U.S., which, together with its high concentration of vitamin C, contributes substantially to its total antioxidant capacity [6,7]. Fruit juice consumers also tend to consume more whole fruit [8,9], which is one indicator of a healthier diet. Fruit juice itself is an important contributor to fruit intake, providing approximately one-third of the total fruit intake across all ages [10]. This is particularly salient because overall fruit intake, including fruit juice, is below the recommendations among all age groups except young children [10].

Despite the nutritional benefits of fruit juice, there have been concerns regarding its potential contribution to childhood obesity, leading to authorities recommending limiting its consumption. The American Academy of Pediatrics and the U.S. Department of Agriculture recommend limiting fruit juice intake to contribute no more than half the daily total fruit intake—equal to 8 fl oz. per day for 7–18 year old children [11]—because juice offers no nutritional advantage over whole fruit, lacks in dietary fiber and can potentially contribute excess calories when consumed in excess [10,11]. While the recommendations of moderate fruit juice intake are reasonable, particularly for those at risk of becoming overweight/obese, restricting fruit juice intake reduces the intake of healthful nutrients, and so a careful balance must be struck between moderating caloric intake and restricting the intake of a source of healthful vitamins, minerals and antioxidants. In light of these recommendations, the literature regarding fruit juice’s association with adiposity is mixed. Several epidemiologic studies have identified fruit juice as a risk factor for obesity and excess weight gain in children aged 10 years and younger [12,13,14,15]. However, others have shown that fruit juice does not contribute to weight gain or obesity in young children or adolescents when controlling for relevant factors such as age, race or total energy intake [3,16,17,18]. Additionally, orange juice specifically was not associated with adiposity in children 18 years old and younger [4,19]. A possible explanation for the lack of consistency in previous findings may be due to the failure to explicitly measure the intake of 100% fruit juice [13,20,21,22]. This results in a misclassification bias where 100% fruit juice is potentially categorized with other sugar-sweetened beverages such as fruit-flavored soft drinks, diluting the associations of 100% fruit juice. Furthermore, there are a limited number of epidemiologic studies with large sample sizes that investigate fruit juice’s effect on health in children and adolescents. Therefore, the objective of this study was to evaluate the associations between OJ consumption and dietary, lifestyle and anthropometric characteristics in a large cohort of U.S. children and adolescents.

## 2. Materials and Methods

### 2.1. Study Population

The Growing Up Today Study (GUTS) is a cohort study that began enrollment in its first phase (GUTS I) in 1996, which included boys and girls age 9–14 years old, and was followed by a second phase (GUTS II) in 2004, which included boys and girls age 9–16 years old. The cohort recruited participants from all 50 U.S. states, and participants were offspring of participants in the Nurses’ Health Study II (NHS II). Mothers provided informed consent and the children assented by completing baseline questionnaires. The collective enrollment of the two phases of GUTS was 27,805 (16,882 in GUTS I and 10,923 in GUTS II). The current study included children who responded to baseline questionnaires in 1996 (GUTS I) or 2004 (GUTS II). As the baseline characteristics were similar between cohorts, data from both cohorts were combined. Children were excluded if their baseline questionnaires were missing information on OJ consumption, height or weight, physical activity, screen time, fruit consumption or non-starchy vegetable consumption, or if they had an extremely low BMI (<12 kg/m^2^), yielding a final analytic cohort of 26,554 participants.

### 2.2. OJ Intake

Baseline questionnaires included a semi-quantitative food frequency questionnaire (FFQ) designed for and validated in older children and adolescents [23,24]. The FFQ performed comparably with a FFQ designed for adults, with a mean correlation of r = 0.54 for nutrients from the FFQ compared with three 24-h recalls. OJ consumption was determined from the FFQ question asking children how often during the past year they consumed orange juice. Response options consisted of the following: never/less than 1 glass per month, 1–3 glasses per month, 1 glass per week, 2–6 glasses per week, 1 glass per day or more than 1 glass per day. The weekly number of glasses of OJ consumed was calculated by converting these responses to a continuous variable. When reported OJ consumption was designated as a serving range, the median of the response range was used (e.g., “2–6 glasses per week” was assumed to be 4 glasses per week). Children reporting consumption of more than 1 glass per day were assumed to consume 2 glasses per day. OJ non-consumers were defined as those that responded with the lowest consumption category of never/less than 1 glass per month, while OJ consumers were those that responded with any of the other options. The FFQ was structured such that questions pertaining to the intakes of soda, diet soda and other sugar-sweetened beverages preceded the question pertaining to the intake of OJ; the question pertaining to the intakes of fruit juices other than OJ followed. This structuring increased the likelihood that respondents interpreted the OJ question as referring to either freshly squeezed OJ from oranges or a commercially prepared, 100% OJ drink.

### 2.3. Anthropometric Data

The participants self-reported height and weight in the questionnaires using specific measuring instructions. Participants were recommended to seek assistance from their mothers, who biennially self-reported their own weights for the NHS II [25]. Self-reported height and weight were validated among preadolescents and adolescents (weight r ≥ 0.87, height r ≥ 0.82) [26,27,28]. BMI was calculated using the self-reported height and weight data, and z-scores were calculated for height-for-age (HAZ) and BMI-for-age. A BMI-for-age percentile was used to categorize children’s weight statuses according to the cutoffs defined by the U.S. Centers for Disease Control and Prevention [29]: underweight = BMI-for-age < 5th percentile, normal weight = 5th ≤ BMI-for-age ≤ 85th, overweight = 85th < BMI-for-age ≤ 95th, obese = BMI-for-age > 95th.

### 2.4. Other Variables

Fruit intake was calculated by summing children’s FFQ responses for consumption of the following nine items: raisins, grapes, bananas, apples/applesauce, cantaloupe/melons, pears, oranges/grapefruits, strawberries and peaches/plums/apricots. One-hundred percent fruit juice (of any type) was not included in fruit intake. Non-starchy vegetable consumption was assessed as the sum of responses to intake of the following thirteen items: tomatoes, string beans, broccoli, mixed vegetables, spinach, collard greens/kale, peppers, zucchini/summer squash/eggplant, cooked carrots, raw carrots, celery, lettuce and coleslaw. The number of servings of fruits and vegetables was derived from the categorical responses from the FFQ and converted to a continuous variable of daily servings. Total energy intake was derived from the FFQ. Since the volume of a glass of OJ was undefined in the FFQ, we calculated total energy intake excluding OJ by assuming each glass of OJ reported was 6 fl oz and provided 85 kcal. We made this assumption based off previously measured OJ intake in children and adolescents [5] and assumed a reasonable volume of OJ that a child is likely to consume in a single serving.

Physical activity was calculated by multiplying the hours spent per week engaged in activities of moderate to vigorous intensity, defined as 4 metabolic equivalent of task (MET) or greater [30], by the activities’ MET scores. Activities meeting this level of intensity were identified based on their MET in the Compendium of Physical Activities [30]. The average number of weekly hours spent in each qualifying activity was multiplied by its MET, and the products were summed to calculate weekly MET hours. In the 1996 survey for GUTS I, questions pertaining to physical activity asked respondents about the number of hours per week they had participated in sports or other physical activities over the past 12 months. In the 2004 survey for GUTS II, questions for each activity were further divided by season (e.g., “how many hours per week did you participate in soccer during the fall?”), which likely increased the accuracy of estimates over the past 12 months.

Screen time was calculated by summing the weekly time spent watching television, playing video games, watching videos/movies and using the computer/internet (available in GUTS II only) as reported by the children in the questionnaire.

### 2.5. Statistical Analyses

All statistical analyses were conducted using SAS version 9.4. Average values for anthropometric measurements and dietary and lifestyle factors were calculated along with standard deviations for boys and girls separately, and in both the combined GUTS cohorts and within groups of varying OJ consumption levels. A trend to determine if the OJ consumption category was linearly related to these variables was assessed using multiple comparisons based on linear contrast. Odds ratios (ORs) and 95% confidence intervals (CIs) for being obese or overweight/obese were calculated for each OJ consumption category using low/non-consumers (<1 glass OJ per month) as a reference. ORs were calculated after adjustment for potential confounders including cohort (GUTS I vs GUTS II), age, race and total energy intake excluding OJ (Model 1), then additionally adjusted for physical activity and screen time (Model 2). Linear trend testing was again used to examine whether the OJ category was linearly related to the odds of obesity or obesity/overweight. *p*-values < 0.05 were considered to be statistically significant.

## 3. Results

### 3.1. Baseline Characteristics

The characteristics of the GUTS I and GUTS II cohorts at baseline are summarized in Table 1. The majority of participants were non-Hispanic white and normal weight status. The mean total daily caloric intake was 2294 ± 719 kcal for boys and 2052 ± 647 kcal for girls in GUTS I, and 2327 ± 757 kcal for boys and 1957 ± 658 kcal for girls in GUTS II. The majority of participants consumed at least 1 glass of OJ per month and over a third of participants consumed at least 2–6 glasses of OJ per week. Across both cohorts and genders, fewer than 7% of participants reported consuming more than 1 glass of OJ daily. Moderate/vigorous physical activity and screen time appeared to differ between cohorts, which may reflect changes in survey questions and response options between 1996 and 2004.

### 3.2. Demographic and Anthropometric Characteristics of Participants by OJ Consumption

In the combined cohort analyses, boys consuming more OJ tended to be older (*p*-trend < 0.0001) but girls’ ages did not significantly differ by OJ consumption (*p*-trend = 0.1421) (Table 2). Boys and girls consuming more OJ also tended to be taller, as measured by both height (boys *p*-trend < 0.0001, girls *p*-trend = 0.0003) and HAZ (boys *p*-trend = 0.0001, girls *p*-trend = 0.0031). There were also significant associations between OJ consumption and prevalence of obesity (boys *p*-trend < 0.0001, girls *p*-trend = 0.0016) and overweight/obesity (boys *p*-trend < 0.0001, girls *p*-trend = 0.0006). BMI and BMI-for-age percentile did not significantly differ by OJ consumption in either boys or girls.

### 3.3. Dietary and Lifestyle Characteristics of Participants by OJ Consumption

Intakes of total energy, total energy excluding OJ, fruits and non-starchy vegetables were positively associated with OJ consumption in boys and girls (*p*-trends < 0.0001) (Table 3). Additionally, boys and girls consuming more OJ tended to engage in more moderate/vigorous physical activity (*p*-trends < 0.0001). Screen time significantly decreased with OJ consumption in boys but did not significantly differ in girls.

### 3.4. Likelihood of Obesity and Overweight by OJ Consumption

Since the prevalence of obesity and overweight/obesity was found to be associated with OJ consumption in our previous analysis, we further explored the association by evaluating the likelihood of being obese or overweight/obese using a linear trend. In boys, greater OJ consumption was significantly associated with reduced odds of being obese or overweight/obese after adjusting for cohort, age, race and total energy intake excluding OJ (Model 1, Table 4). This trend remained significant after additionally adjusting for moderate/vigorous physical activity and screen time. Importantly, although there was a decreasing linear trend in the prevalence of obesity and overweight/obesity, there was no prevalence association for any individual category of OJ consumption (the 95% CIs crossed over the referent value). In girls, there were no significant differences in the odds of being obese or overweight/obese by OJ consumption categories. These results indicate that OJ consumption was not associated with an increased prevalence of obesity or overweight/obesity and was, in fact, negatively associated in boys.

## 4. Discussion

In this cross-sectional evaluation of children and adolescents from the GUTS I and GUTS II cohorts, OJ consumption was associated with a lower prevalence of obesity and overweight/obesity in boys, but had no association in girls. Interestingly, this lower prevalence persisted in spite of increased total energy intake excluding OJ by OJ consumption category, which might cause one to expect the opposite results. This suggests that OJ consumption was also linked to higher energy requirements—as indicated by physical activity, it is likely that those consuming greater amounts of OJ have a greater energy demand due to sports or other moderate/vigorous activities. Other studies have found that OJ consumption was not associated with other metrics of adiposity including BMI and waist circumference [4,19]. With regards to other types of fruit juice, total fruit juice intake in the preadolescent or adolescent age groups was similarly not associated with adiposity [9,23,24,31]. The reasons underlying the disparity in prevalence of overweight/obesity and obesity between boys and girls remains unclear and deserves further investigation, but may in part be due to gender-specific differences in the reliability of self-reported weight in young adolescents. While the correlation between self-reported and actual weight in young adolescents is high, girls tend to provide slightly less reliable self-reported weight data than boys [26]. Still, the degree to which boys and girls differ in providing reliable self-reported weight data is minor and not expected to be the sole contributor to the difference observed in our analysis. Overall, these results substantiate claims that moderate fruit juice intake can be included in dietary recommendations to increase fruit intake without increasing adiposity.

There was a slight difference in both height and HAZ between OJ consumers; however, due to the limitations of cross-sectional analyses, this study cannot determine the direction of the association. The relationship between 100% juice intake and height in adolescents has scarcely been described, but has previously been shown to have no association with height in younger children [14,32]. Future studies will need to assess changes in height growth in relation to OJ intake to best evaluate the effects of OJ intake on skeletal development.

Greater OJ consumption was associated with greater intakes of other fruits as well as non-starchy vegetables. While the present study did not evaluate micronutrient intakes as an outcome, others have shown that 100% juice consumption is positively associated with intakes of antioxidants, polyphenols, vitamins and minerals such as calcium, potassium, folate, magnesium and vitamins A, C and D [4,5,17]. Findings from other studies in children 18 years and younger also support that 100% juice intake is correlated with whole fruit intake; however, they observed no association with vegetable intake [4,9,17]. The difference in findings with vegetable intake may be related to the varying definitions of vegetables between studies. Starchy vegetables such as peas, corn and potatoes are commonly included in the category of vegetables in dietary assessments. However, since starchy vegetables contain noticeably more calories than non-starchy vegetables, they were excluded from our definition. Our aim was to use vegetable intake as an indicator of diet quality, which considers the quantity of beneficial nutrients against its caloric content. Ramsay et al. reported that children consuming more non-starchy vegetables but not white potatoes scored higher on the Healthy Eating Index 2010, indicating that vegetable intake is associated with diet quality [25]. Our results suggest that greater OJ consumption is linked with greater non-juice fruit and non-starchy vegetable intake and thus better diet quality. Given the positive association between OJ consumption and total energy intake excluding OJ, the increased fruit and vegetable intake may simply be a product of eating more food rather than indicating greater per-calorie consumption of fruits and vegetables. The lack of increase in overweight or obesity prevalence and BMI-for-age percentile across levels of OJ consumption suggests that the caloric intake of those consuming more OJ is likely not in excess. Nonetheless, increased crude intake of fruits and vegetables contributes greater amounts of health-promoting micronutrients and fiber.

With regards to lifestyle behaviors, greater consumption of OJ was associated with greater physical activity, and screen time in boys. In addition to OJ [5,19,24], apple juice [24] has been reported to be positively associated with physical activity. As physical activity and screen time are not necessarily mutually exclusive, these results suggest that participants drinking more OJ likely committed more non-screen time to physical tasks or engaged in more physically demanding activities.

To our knowledge, this study is the largest cross-sectional evaluation of the associations between 100% OJ consumption and dietary, anthropometric and lifestyle characteristics in U.S. adolescents and older children. In fact, the sample size used in this study was about quadruple the sample size used by others [4,19] who have similarly investigated the associations between OJ consumption and various health-related characteristics in children and adolescents. The benefit of a large sample size is the enhanced precision of estimates and reliability of results. Furthermore, the observed associations with OJ are likely accurate, as the FFQ specified consumption of OJ that was separate from consumption of other 100% fruit juices as well as soda and other fruit-flavored drinks that contain added sugar. This distinction reduces misclassification errors that may result from OJ from being categorized with other fruit-flavored beverages, and ensures our analysis specifically relates to 100% OJ intake. All participants were the children of nurses who participated in the NHS II, so the accuracy of the self-reported information is likely to be enhanced. However, this also means that the GUTS participants were not entirely representative of the U.S. population. There was also a relative lack of racial/ethnic diversity, which may have limited the generalizability of the findings. Another limitation is that temporal relations and causality cannot be determined, as this was a cross-sectional study. Finally, our study relied on self-reported weight and height, which introduces a potential information bias. However, self-reported weight and height have previously been validated in this age group [26,27,28].

## 5. Conclusions

Findings from this cross-sectional evaluation of 100% OJ consumption in U.S. adolescents suggest that OJ consumption is associated with healthier dietary behaviors and greater physical activity, and negatively associated with obesity and overweight/obesity prevalence in boys. Given the nutritional benefits and its contribution to overall fruit intake, OJ should not be disregarded as a healthful beverage when consumed in moderation. Future longitudinal studies are needed to clarify the association of OJ consumption with changes in weight status and health-related outcomes.

## Figures and Tables

**Table 1:** Baseline characteristics of GUTS I and II participants (mean ± SD, or %).

Characteristics	GUTS I		GUTS II	
	Boys	Girls	Boys	Girls
N	7633	8799	4581	5541
Age (y)	12.0 ± 1.6	12.1 ± 1.6	13.0 ± 1.9	13.1 ± 1.9
Non-Hispanic white	93.2	93.2	94.1	92.4
Height (cm)	150.9 ± 13.1	149.8 ± 11.8	159.4 ± 14.2	155.9 ± 10.3
HAZ	0.07 ± 1.11	−0.08 ± 1.09	0.05 ± 1.13	−0.08 ± 1.07
BMI (kg/m^2^)	19.3 ± 3.6	19.2 ± 3.6	20.5 ± 3.9	20.3 ± 3.8
BMI z-score	0.24 ± 1.06	0.10 ± 1.01	0.30 ± 1.06	0.16 ± 0.97
BMI-for-age percentile	57.6 ± 29.3	53.4 ± 28.9	58.9 ± 29.2	55.4 ± 27.7
Obese ^1^	9.20	5.67	10.0	5.78
Overweight ^1^	14.4	12.6	15.3	12.3
Normal weight ^1^	72.1	77.1	70.7	78.1
Underweight ^1^	4.27	4.67	3.99	3.79
Moderate/vigorous physical activity (MET-hrs/wk) ^2^	119.5 ± 74.3	88.6 ± 61.1	92.8 ± 67.6	68.0 ± 50.8
Screen time (hrs/wk) ^3^	31.8 ± 17.3	25.4 ± 14.8	24.4 ± 17.0	19.3 ± 14.0
Total energy intake (kcal/d)	2294 ± 719	2052 ± 647	2327 ± 757	1957 ± 658
Total energy intake excluding OJ (kcal/d)	2255 ± 708	2019 ± 638	2295 ± 747	1929 ± 648
Fruits (svg/d) ^4^	0.96 ± 0.75	1.04 ± 0.77	0.93 ± 0.76	1.00 ± 0.76
Non-starchy vegetables (svg/d) ^4^	0.96 ± 0.78	1.09 ± 0.85	0.98 ± 0.81	1.13 ± 0.88
OJ intake (glasses/wk) ^4^	3.22 ± 1.47	3.04 ± 1.45	2.65 ± 3.38	2.23 ± 3.04
Never/less than 1 glass per month	13.9	16.7	19.7	25.3
1–3 glasses per month	23.6	25.3	23.8	25.1
1 glass per week	17.5	18.1	17.3	15.7
2–6 glasses per week	24.2	21.8	23.1	20.5
1 glass per day	14.2	13.2	11.5	10.6
More than 1 glass per day	6.75	4.86	4.54	2.83

^1^ Categorization on the basis of cutoffs defined by U.S. Centers for Disease Control and Prevention (2016 update). Obese: BMI-for-age ≥ 95th percentile; overweight: 95th > BMI-for-age ≥ 85th percentile; normal weight: 85th > BMI-for-age ≥ 5th percentile; underweight: BMI-for-age < 5th percentile. ^2^ Total metabolic equivalent (MET) hours per week calculated by summing the time spent participating in various sports, games and intentional exercise (not including activities with MET values < 4 such as walking and “playing outdoors” in GUTS I). ^3^ Including television and VCR/DVD videos, video games and computer/internet. ^4^ Glasses consumed were calculated from the categorical response options from the food frequency questionnaire. Percentages may not add up to 100.0 due to rounding. GUTS, Growing Up Today Study; HAZ, height-for-age z-score; OJ, orange juice.

**Table 2:** Baseline demographic and anthropometric characteristics of the GUTS I and II participants by level of 100% OJ consumption (mean ± SD, or %).

Characteristics	Never or <1 Glass Per Month	1–3 Glasses per Month	1–6 Glasses per Week	1 Glass per Day	>1 Glass per Day	*p*-Trend *
Boys						
N	1962	2890	5030	1609	723	
Age (y)	12.3 ± 1.8	12.5 ± 1.8	12.6 ± 1.8	12.5 ± 1.8	12.7 ± 1.8	<0.0001
Non-Hispanic white	93.1	93.6	93.6	93.9	93.4	0.4612
Height (cm)	152.2 ± 14.2	153.7 ± 13.9	154.8 ± 14.1	154.4 ± 14.0	156.0 ± 14.4	<0.0001
HAZ	−0.03 ± 1.12	0.05 ± 1.13	0.08 ± 1.11	0.13 ± 1.10	0.12 ± 1.11	0.0001
BMI (kg/m^2^)	19.6 ± 3.9	19.9 ± 3.9	19.8 ± 3.7	19.5 ± 3.5	19.6 ± 3.5	0.2019
BMI z-score	0.23 ± 1.12	0.31 ± 1.08	0.28 ± 1.04	0.21 ± 1.05	0.20 ± 1.00	0.1019
BMI-for-age percentile	56.9 ± 30.3	59.3 ± 29.7	58.4 ± 28.9	56.9 ± 28.7	56.4 ± 28.0	0.1992
Obese ^1^	10.2	10.9	9.30	7.83	6.78	<0.0001
Overweight/obese ^1^	24.6	26.9	24.2	21.6	19.1	<0.0001
Girls						
N	2875	3617	5518	1745	585	
Age (y)	12.2 ± 1.9	12.2 ± 1.9	12.2 ± 1.9	12.2 ± 1.9	12.3 ± 1.8	0.1421
Non-Hispanic white	93.0	92.6	93.0	94.0	90.3	0.8409
Height (cm)	151.3 ± 11.6	152.1 ± 11.4	152.5 ± 11.7	152.3 ± 11.8	153.2 ± 11.9	0.0003
HAZ	−0.16 ± 1.09	−0.07 ± 1.09	−0.06 ± 1.07	−0.04 ± 1.06	−0.03 ± 1.13	0.0031
BMI (kg/m^2^)	19.6 ± 3.8	19.8 ± 3.9	19.6 ± 3.6	19.3 ± 3.4	19.8 ± 3.8	0.4976
BMI z-score	0.13 ± 1.03	0.17 ± 1.01	0.11 ± 0.98	0.05 ± 0.98	0.15 ± 0.99	0.4011
BMI-for-age percentile	54.6 ± 29.0	55.3 ± 28.7	53.7 ± 28.2	52.2 ± 28.4	54.8 ± 28.1	0.3226
Obese ^1^	6.05	7.22	5.00	4.00	6.67	0.0016
Overweight/obese ^1^	19.3	20.0	17.2	15.3	19.3	0.0006

^1^ Categorization on the basis of cutoffs defined by U.S. Centers for Disease Control and Prevention (2016 update). Obese: BMI-for-age ≥ 95th percentile; overweight/obese: BMI-for-age≥ 85th percentile. * For categorical variables, *p*-value based on Cochran–Mantel–Haenszel statistics; for continuous variables, *p*-value represents the trend of the linear relationship assessed from multiple comparisons based on linear contrast. GUTS, Growing Up Today Study; HAZ, height-for-age z-score; OJ, orange juice.

**Table 3:** Baseline dietary and lifestyle characteristics of the GUTS I and II participants by level of 100% OJ consumption (mean ± SD).

Characteristics	Never or <1 Glass per Month	1–3 Glasses per Month	1–6 Glasses per Week	1 Glass per Day	>1 Glass per Day	*p*-Trend*
Boys						
N	1962	2890	5030	1609	723	
Total energy intake (kcal/d)	2089 ± 723	2078 ± 674	2380 ± 701	2535 ± 709	2782 ± 776	<0.0001
Total energy intake excl. OJ (kcal/d)	2089 ± 723	2072 ± 674	2347 ± 699	2450 ± 709	2612 ± 776	<0.0001
Fruits (svg/d)^1^	0.67 ± 0.61	0.72 ± 0.58	1.02 ± 0.71	1.25 ± 0.87	1.44 ± 1.04	<0.0001
Non-starchy vegetables (svg/d) ^1^	0.73 ± 0.69	0.74 ± 0.64	1.04 ± 0.77	1.28 ± 0.93	1.29 ± 0.97	<0.0001
Moderate/vigorous physical activity (MET*hrs/wk) ^2^	92.3 ± 67.4	102.0 ± 68.5	114.1 ± 73.3	118.9 ± 76.1	132.8 ± 83.0	<0.0001
Screen time (hrs/wk) ^3^	29.2 ± 18.1	30.0 ± 17.7	29.1 ± 17.1	27.3 ± 17.2	28.7 ± 17.8	0.0201
Girls						
N	2875	3617	5518	1745	585	
Total energy intake (kcal/d)	1828 ± 618	1848 ± 605	2094 ± 627	2275 ± 658	2450 ± 720	<0.0001
Total energy intake excl. OJ (kcal/d)	1828 ± 618	1842 ± 605	2062 ± 625	2190 ± 658	2280 ± 720	<0.0001
Fruits (svg/d)^1^	0.79 ± 0.66	0.83 ± 0.63	1.10 ± 0.72	1.35 ± 0.91	1.62 ± 1.14	<0.0001
Non-starchy vegetables (svg/d) ^1^	0.87 ± 0.75	0.93 ± 0.76	1.20 ± 0.84	1.39 ± 0.95	1.58 ± 1.16	<0.0001
Moderate/vigorous physical activity (MET*hrs/wk) ^2^	70.5 ± 54.1	79.3 ± 60.3	88.5 ± 61.7	96.2 ± 66.7	105.1 ± 73.6	<0.0001
Screen time (hrs/wk) ^3^	22.7 ± 15.0	23.9 ± 15.4	22.9 ± 14.5	22.0 ± 14.4	22.6 ± 14.4	0.1252

^1^ Servings were calculated from the categorical response options from the FFQ. ^2^ Total metabolic equivalent hours per week calculated by summing the time spent participating in various sports, games and intentional exercise (not including activities with MET values < 4 such as walking and “playing outdoors” in GUTS I). ^3^ Including television and VCR/DVD videos, video games and computer/internet. * *p*-value represents the trend of the linear relationship assessed from multiple comparisons based on linear contrast. GUTS, Growing Up Today Study; OJ, orange juice.

**Table 4:** Adjusted odds ratios for obesity and overweight of GUTS I and II participants by level of 100% OJ consumption (OR and 95% CI).

Weight Status	Never or <1 Glass per Month	1–3 Glasses per Month	1–6 Glasses per Week	1 Glass per Day	>1 Glass per Day	*p*-Trend *
Boys						
N	1962	2890	5030	1609	723	
Obese ^1^, N	201	315	468	126	49	
Model 1	Ref (1.0)	1.10 (0.91, 1.32)	0.97 (0.81, 1.16)	0.82 (0.65, 1.04)	0.73 (0.53, 1.02)	0.0028
Model 2	Ref (1.0)	1.12 (0.93, 1.36)	1.04 (0.87, 1.25)	0.91 (0.72, 1.15)	0.83 (0.59, 1.15)	0.0430
Overweight/obese ^1^, N	483	776	1217	347	138	
Model 1	Ref (1.0)	1.15 (1.01, 1.31)	1.06 (0.94, 1.20)	0.93 (0.79, 1.09)	0.84 (0.67, 1.04)	0.0019
Model 2	Ref (1.0)	1.17 (1.02, 1.33)	1.11 (0.98, 1.26)	1.00 (0.85, 1.18)	0.91 (0.73, 1.13)	0.0403
Girls						
N	2875	3617	5518	1745	585	
Obese ^1^, N	174	261	276	69	39	
Model 1	Ref (1.0)	1.23 (1.01, 1.50)	0.87 (0.72, 1.07)	0.70 (0.52, 0.94)	1.24 (0.86, 1.79)	0.1235
Model 2	Ref (1.0)	1.24 (1.01, 1.51)	0.95 (0.78, 1.16)	0.80 (0.60, 1.07)	1.46 (1.01, 2.11)	0.7535
Overweight/obese ^1^, N	556	723	947	267	113	
Model 1	Ref (1.0)	1.06 (0.93, 1.20)	0.93 (0.83, 1.05)	0.83 (0.71, 0.98)	1.15 (0.91, 1.44)	0.4000
Model 2	Ref (1.0)	1.06 (0.94, 1.20)	0.98 (0.87, 1.11)	0.91 (0.78, 1.08)	1.24 (1.02, 1.62)	0.5607

^1^ Categorization on the basis of cutoffs defined by U.S. Centers for Disease Control and Prevention (2016 update). Obese: BMI-for-age ≥ 95th percentile; Overweight/obese: BMI-for-age ≥ 85th percentile. Model 1: adjusted for cohort, age, race and total energy intake excluding OJ. Model 2: adjusted for cohort, age, race, total energy intake excluding OJ, moderate/physical activity and screen time. * *p*-value represents the trend of the linear relationship assessed from multiple comparisons based on linear contrast. CI, confidence interval; GUTS, Growing Up Today Study; OJ, orange juice; OR, odds ratio.
